# ConReg-R: Extrapolative recalibration of the empirical distribution of p-values to improve false discovery rate estimates

**DOI:** 10.1186/1745-6150-6-27

**Published:** 2011-05-20

**Authors:** Juntao Li, Puteri Paramita, Kwok Pui Choi, R Krishna Murthy Karuturi

**Affiliations:** 1 Computational & Mathematical Biology, Genome Institute of Singapore, 60 Biopolis Street, Singapore 138672, Singapore; 2 Department of Statistics and Applied Probability, National University of Singapore, 6 Science Drive 2, Singapore 117546, Singapore

## Abstract

**Background:**

False discovery rate (FDR) control is commonly accepted as the most appropriate error control in multiple hypothesis testing problems. The accuracy of FDR estimation depends on the accuracy of the estimation of p-values from each test and validity of the underlying assumptions of the distribution. However, in many practical testing problems such as in genomics, the p-values could be under-estimated or over-estimated for many known or unknown reasons. Consequently, FDR estimation would then be influenced and lose its veracity.

**Results:**

We propose a new extrapolative method called *Constrained Regression Recalibration *(ConReg-R) to recalibrate the empirical p-values by modeling their distribution to improve the FDR estimates. Our ConReg-R method is based on the observation that accurately estimated p-values from true null hypotheses follow uniform distribution and the observed distribution of p-values is indeed a mixture of distributions of p-values from true null hypotheses and true alternative hypotheses. Hence, ConReg-R recalibrates the observed p-values so that they exhibit the properties of an ideal empirical p-value distribution. The proportion of true null hypotheses (*π*_0_) and FDR are estimated after the recalibration.

**Conclusions:**

ConReg-R provides an efficient way to improve the FDR estimates. It only requires the p-values from the tests and avoids permutation of the original test data. We demonstrate that the proposed method significantly improves FDR estimation on several gene expression datasets obtained from microarray and RNA-seq experiments.

**Reviewers:**

The manuscript was reviewed by Prof. Vladimir Kuznetsov, Prof. Philippe Broet, and Prof. Hongfang Liu (nominated by Prof. Yuriy Gusev).

## Background

In high-throughput biological data analysis, multiple hypothesis testing is employed to address certain biological problems. Appropriate tests are chosen for the data, and the p-values are then computed under some distributional assumptions. Due to the large number of tests performed, error rate controls (which focus on the occurrence of false positives) are commonly used to measure the statistical significance. False discovery rate (FDR) control is accepted as the most appropriate error control. Other useful error rate controls include conditional FDR (cFDR) [1], positive FDR (pFDR) [2] and local FDR (lFDR) [3] which have similar interpretations as that of FDR. However, appropriate FDR estimation depends on the precise p-values from each test and the validity of the underlying assumptions of the distribution.

The p-values from multiple hypothesis testing, for *n *hypotheses, can be described by a mixture model *g*(*p*) (1) with two components: one component *g*_0_(*p*) originates from true null hypotheses and follows uniform distribution *U*(0, 1) [4], and the other component *g*_1_(*p*) results from true alternative hypotheses and follows a distribution confined to the p-values close to 0 [5]. The mixing parameter, *π*_0_, is the proportion of true null hypotheses in the data. More precisely,(1)

where *g*_0_(*p*) = 1 denotes the probability density function of a uniform distribution over (0, 1) and *g*_1_(*p*) *≈ *0 for *p *close to 1. Therefore, *g*(*p*) will be close to a constant (i.e. *π*_0_) for *p *close to 1.

FDR in multiple hypothesis testing for a given p-value threshold *α *is estimated as

*π*_0 _can be estimated from this mixture model in equation (1) as [2]

where *β *is typically chosen to be 0.25, 0.5 or 0.75. These estimates are reasonable under the uniform distribution assumption of a component in this mixture model [6].

However, in many applied testing problems, the p-values could be under-estimated or over-estimated for many known or unknown reasons. The violation of p-value distribution assumptions may lead to inaccurate FDR estimation. There are many factors influencing FDR estimation in the analysis of high-throughput biological data such as microarray and sequencing studies. Dependence among the test statistics is one of the major factors [7,8]. Usually in microarray data, there are many groups of genes having similar expression patterns and the test statistics (for example, t-statistic) are not independent within one group. The global effects in the array may also influence the dependence in the data. For example, batch and cluster effects [9,10] always occur in the experiments and sometimes they may be the major cause of incorrectly estimated FDR.

Further, due to the "large *p*, small *n*" problem [11] for the gene expression data, some parameters such as mean and variance for each gene cannot be well estimated, or the test assumptions are not satisfied or the distribution of the statistic under null hypotheses may not be accurate. Therefore, many applied testing methods modified the standard testing methods (for example, modifying t-statistic to moderated t-statistic [12]) to increase their usability. As the modified test statistics only approximately follow some known distribution, the approximate p-value estimation may influence the FDR estimation. Resampling strategies may better estimate the underlying distributions of the test statistics. However, due to small sample size and data correlation, the limited number of permutations and resampling bias [13] also influence the FDR estimation.

To address the above problems, we propose a novel extrapolative recalibration procedure called *Constrained Regression Recalibration *(ConReg-R) which models the empirical distribution of p-values in multiple hypothesis testing and recalibrates the imprecise p-value calculation to better approximated p-values to improve the FDR estimation. Our approach focuses on p-values as the p-values from true null hypotheses are expected to follow the uniform distribution and the interference from the distribution of p-values from alternative hypotheses is expected to be minimal towards p = 1. In contrast, the estimation of the empirical null distributions of test statistics may not be accurate as their parametric form may not be known beforehand and their accuracy may depend on the data and the resampling strategy used. ConReg-R first maps the observed p-values to predefined uniformly distributed p-values preserving their rank order and estimates the recalibration mapping function by performing constrained polynomial regression to the *k *highest p-values. The constrained polynomial regression is implemented by quadratic programming solvers. Finally, the p-values will be recalibrated using the normalized recalibration function. FDR is estimated using the recalibrated p-values and the  can be determined during ConReg-R procedure. We demonstrate that our ConReg-R procedure can significantly improve the estimation of FDR on simulated data, and also the environmental stress response time course microarray datasets in yeast and a human RNA-seq dataset.

## Methods

Under the null hypotheses, the p-values are uniformly distributed. Hence, ConReg-R first generates the uniformly distributed p-values within [0, 1] range.

### Uniformly distributed p-value generation

Let *p_i _*denotes the p-value of the *i^th ^*test (*i *= 1, ..., *n*), without loss of generality, we assume *p*_1 _≥ *p*_2 _≥ ... ≥ *p*_*n*_. If we choose a suitable *k *<*n *such that the *i^th ^*null hypothesis  is most likely true, then *p*_1_, ..., *p*_*k *_correspond to the order statistics of *k *independent uniformly distributed random variables provided *p_i_*'s *i*(*i *= 1, ..., *k*) are correctly estimated.

Let  denote the ideal p-values under , and suppose  is known.  can be defined as(2)

Therefore,  are uniformly distributed over .

Then(3)

Using (3), (2) becomes(4)

Since *k *is usually large, *k/*(*k *- 1) is almost 1, therefore  in (4) can be approximated as(5)

We can estimate the recalibration function *f*(*·*), to be described below, between  and  and apply it to all input p-values to output the recalibrated p-values,  i.e.(6)

By Stone-Weierstrass theorem [14], polynomial functions can well approximate any continuous function in the interval [0,1]. Therefore we use polynomial regression to estimate the recalibration function *f*(*·*) satisfying appropriate boundary and monotone constraints.

### Constrained Regression Recalibration (ConReg-R)

Let  and *x_i _*= *p_i_*(*i *= 1 ... *k*), and the recalibration polynomial function *f*(·) is defined as follows,(7)

The constraints *f *(0) = 0, *f *(1) = 1 and *f' *(*x*) *>*0 should be imposed to ensure the orders of the p-values remain the same after the transformation. Furthermore, the constraint for either *f" *(*x*) *>*0 or *f" *(*x*) *<*0 indicates the function *f *should also be a monotonic convex or monotonic concave function to deal with the situations with under-estimated or over-estimated p-values separately and helps in good extrapolation. The constraints *f *(0) = 0 and *f *(1) = 1 can be easily met by scaling and shifting the regression function. Therefore, the regression function only depends on the other two constraints which can be combined into one constraint during the regression procedure.

Quadratic programming (QP) [15] is employed to estimate the regression function as follows: Let **y **= (*y*_1_*, ..., y_k_*)*^T^*, *β *= (*β*_0_, ..., *β*_*t*_)^*T *^and

Equation (7) can be rewritten more succinctly as(8)

and the constrains for the first and second order derivatives of *f *(*X*) will be *Aβ *≥ **b **where **b **= (0, ..., 0)*^T ^*is a 2*l *× 1 vector and

is a 2*l *× (*t *+ 1) matrix, where *a*_1_, ..., *a_l _*are *l *randomly generated numbers following *U*(0, 1) to guarantee

this constraint is valid in (0, 1), and *c *is chosen to be 0 (or 1) if *f *is desired to be convex (or concave respectively).

The least squares procedure for (8) will minimize(9)

Minimizing (9) under *Aβ *≥ **b **is equivalent to minimizing(10)

under *Aβ *≥ **b**, where *Q *= *X^T ^X *and **q **= -*X^T ^***y**. Therefore, the constrained polynomial regression problem can be reformulated as a quadratic programming problem.

#### Two further modifications

We use QuadProg package in *R *to solve the quadratic programming problem [16]. Due to floating point errors [17], *Q *= *X^T ^X *tends to be positive semidefinite instead of being positive definite. To get around this, we add a sufficiently small positive value (*λ *= 10^-10^) to the diagonal of *Q *to guarantee

*Q' *= *Q *+ *λ***I**_*t*+1 _is positive definite and *Q' *replaces *Q *in (10).

Furthermore, the polynomial function may not accurately fit the data due to the limitation of the polynomial maximal power (usually set the maximal power *t *= 10). We can add the fraction of the power (i.e. a non-integer power) to increase the accuracy of the fit. For example, let , where *m *= 1, 2 or more.

#### Computational procedure

**Figure 1:**
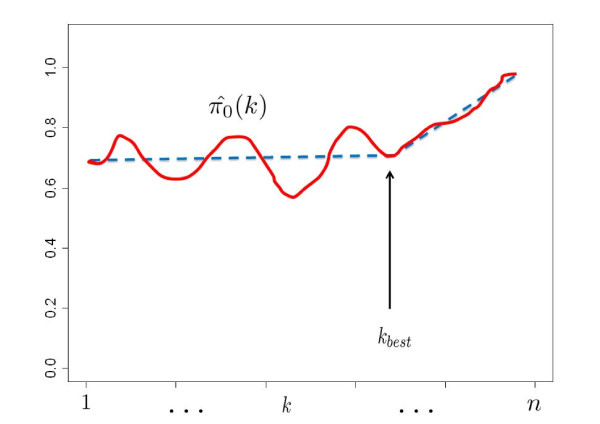
**Illustration of choosing *k_best _*using *k *vs.  plot**. The blue dashed line indicates the ideal *π*_0 _estimated for different choice of *k*. The red curve indicates the actual .

For any given *k*, after applying ConReg-R, the estimation of  and its variation (error) are given by(11)

and(12)

where MAD denotes the median absolute deviation. The final regression function and optimal *k*(*k_best_*) are determined by examining  and  over *k*. Figure 1 illustrates how to choose *k_best _*from the function . Ideally,  is not expected to change over a range of *k *(as shown by the blue dashed line in Figure 1) such that *p*_1_, ..., *p_k _*are most likely to be from null hypotheses. If *k *is too large, *p*_1_, ..., *p_k _*may contain too many p-values from alternate hypotheses and  may be wrongly estimated to be close to 1, in an extreme case if *k *is chosen to be *n *then  = 1. However, the extrapolation in recalibration procedure may be unreliable if only a small number of p-values (i.e. small *k*) are used for the regression and  may fluctuate near the real *π*_0 _(the red curve in Figure 1). Therefore, we aim to choose optimal *k*(*k_best_*) as a trade-off to include just enough p-values from null hypotheses for the regression to achieve good extrapolation. The *k *that gives stable estimate  and the last minimum of  is chosen to be the *k_best_*. The regression function, extrapolation and  corresponding to *k *= *k_best _*are chosen for recalibrating p-values and re-estimating FDR.

The following is the computational procedure for a given  in descending order:

1. For each  (*v *is the interval over *k *and default setting is *v *= [*n/*100]), let .

2. Use equation (5) to compute .

3. Use quadratic programming to obtain regression function *h_k_*, where *c *can be predefined or estimated by checking whether more than half of points for  are above the diagonal (line from origin to (1, 1)) (*c *= 1) or below the diagonal (*c *= 0).

4. Transform *h_k _*to  to satisfy constraints *f_k_*(0) = 0, and *f_k_*(1) = 1.

5. Repeat steps 2-4 for all *k*, and compute the  and  for each *k*. Let *k_best _*be the maximal of *k *which locally minimizes  under the constraint of small , where the cutoff of  and local minimization criteria should be predefined.

6. Choose the final regression function *f *(.) under *k_best _*and output recalibrated p-values.

7. Re-estimate the FDR using recalibrated p-values and .

R-code for ConReg-R is attached as Additional file 1.

## Results

### Dependence simulation

Data dependence is one of the major causes for over- or under-estimated p-values. We simulated an expression data, with dependence, *Z *= (*z_ij _*)(*i *= 1, ..., *n*, *j *= 1, ..., *r*) with *n *(*n *= 10000) genes and *r*(*r *= 10) replicates using the formula as follows,

where *b_i _*denotes the biological effect, *d_ij _*denotes the dependence effect. Set *b_i _*= 1, if *i *≤ *n*(1 - *π*_0_) and *b_i _*= 0 if *i > n*(1 - *π*_0_). *d_i_*. = (1, 1, 1, 0, 0, 0, 0, -1, -1, -1) if  and *d_i_*. = (-1, -1, -1, 0, 0, 0, 0, 1, 1, 1) if ). *ε_ij _~ N*(0, 1) is the background noise.

**Figure 2:**
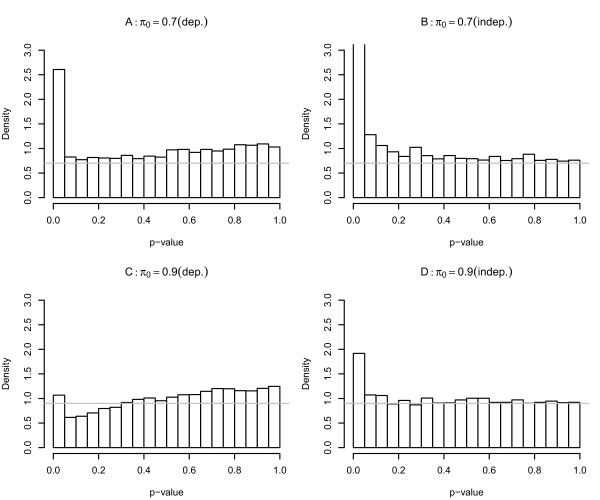
**The density histograms of dependent datasets and independent datasets at *π*_0 _= 0.7 and *π*_0 _= 0.9, and the gray horizontal line indicates the *π*_0 _for each dataset**.

To compare the result, we also simulated a data set with no dependence using the same procedure but with the dependence effect *d_ij _*= 0. One sample t-test was performed to generate p-values. Figure 2 shows the p-value density histograms for *π*_0 _= 0.7 and *π*_0 _= 0.9. As can be seen in the plots B and D in Figure 2, the p-value histograms from independent data have constant frequency for *p *≥ 0.5 and the density near 1 indicates the . However, the p-value histograms from dependent data (the plots A and C in Figure 2) do not have such constant frequency and p-value density increases as p-value increases in the neighborhood of 1. The density near 1 exceeds the respective *π*_0_.

**Figure 3:**
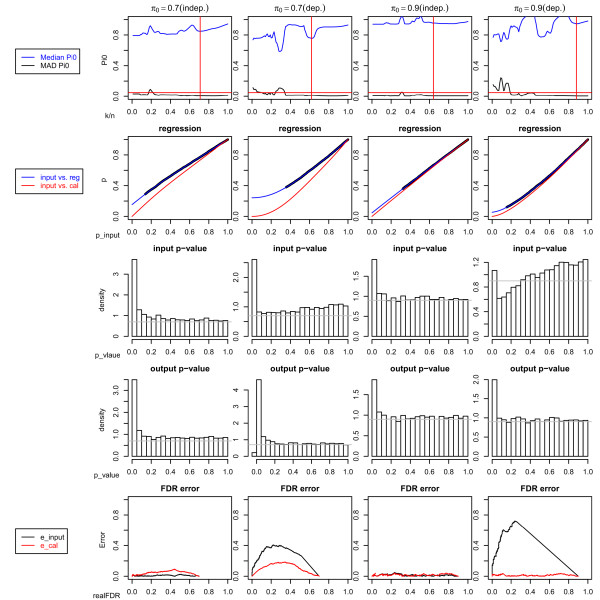
**The procedural steps for the independent and dependent datasets at *π*_0 _= 0.7 and *π*_0 _= 0.9**. The plots in first row show the  and  at different *k/n*. The blue curve indicates  and the black curve indicates , the red horizontal line indicates the cutoff of  (here we used 0.05), the red vertical line indicates the choice of *k/n *at which locally minimized  under  < 0.05 is obtained. The plots in second row show the regression procedure. The black thick curve indicates the , *i *= 1, ..., *k *and the blue curve is the regression line *h_k_*(.), and the red curve is the regression line *f *(.) after transformation. The plots in third and fourth row show the p-value histograms before and after applying ConReg-R and the gray horizontal line indicates the *π*_0_. The plots in last row show the FDR estimation errors between real FDR and the FDR estimated by p-values before (black) and after applying ConReg-R (red).

ConReg-R used the above p-values as input and output the recalibrated p-values. The results are summarized in Figure 3. For the independent data sets, the algorithm chose *k *= 0.71*n *for *π*_0 _= 0.7 and *k *= 0.64*n *for *π*_0 _= 0.9 since it locally minimized  under . The p-values do not significantly change after regression. As such, the regression curves almost overlap with the diagonals, and the input p-value histogram and the output p-value histogram are very similar to each other. The FDR estimation errors (the absolute difference between FDR estimated by p-values and real FDR) also do not significantly change after applying ConReg-R and the estimation of FDR is very close to the real FDR. However, for the dependent data sets, the algorithm chose *k *= 0.62*n *for *π*_0 _= 0.7 and *k *= 0.88*n *for *π*_0 _= 0.9. The regression curves are all below the diagonals and the output p-value histograms after applying ConReg-R appears more like the ones obtained for the independent data. The accuracy of estimated FDR after applying ConReg-R is substantially improved.

**Figure 4:**
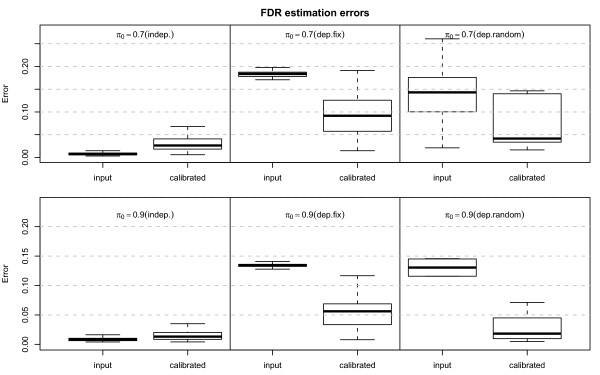
**Boxplots of FDR estimation errors (the mean difference between real FDR and the FDR estimated by p-values) for 100 simulations of independent and dependent datasets at *π*_0 _= 0.7 and *π*_0 _= 0.9 before (input) and after applying ConReg-R (calibrated)**.

To study more complicated dependency situations, we generated dependent datasets with random dependence effect [8] as follows,

where *ρ *is the correlation constant (here we set *ρ *= 0.5) which determines the correlation coefficient between genes. Here *b_i _*denotes the biological effect, and *d_j _*denotes the random dependence effect. Set *b_i _*= 1, if *i ≤ n*(1 *- π*_0_) and *b_i _*= 0 if *i > n*(1 *- π*_0_), and *d_j _~ N*(0, 1). Let *ε_ij _N*(0, 1) be the background noise. The result for *π*_0 _= 0.7 and *π*_0 _= 0.9 are shown in Additional File 2, Figure S1. Similar to the simulations of fixed dependence effect, the estimated FDR after applying ConReg-R is closer to real FDR. The results of our procedure for 100 repeated simulations are summarized in the box-and-whisker plots in Figure 4. As shown in this figure, for the independent data sets, the FDR estimation errors (the mean absolute difference between real FDR and the FDR estimated by p-values using Benjamini-Hochberg method) after applying ConReg-R is slightly higher. However, it is still acceptable since most simulation resulted in errors below 0.05. For the dependent data sets with fixed and random dependence effects, the FDR estimation errors after applying ConReg-R are significantly less than those without applying ConReg-R. The FDR estimation for *π*_0 _= 0.9 is even closer to real FDR after applying ConReg-R compared with the result for *π*_0 _= 0.7 because of more p-values used for regression and less number of p-values for extrapolating in datasets of *π*_0 _= 0.9.

### Combined p-values simulation

In many analyses, more than one dataset are involved and a meta-analysis by combining p-values from different studies or datasets is needed to estimate the overall significance for each gene. For example, (i) to find genes which are significant in at least one experiment, minimal p-values will be of interest; (ii) to identify genes which are significant across all the experiments, the maximal p-values will be of interest; and (iii) in order to detect genes which are significant on average, the product of p-values will be appropriate. The distribution of combined p-values will not be uniform even under true null hypotheses [18]. For currently used meta-analysis methods, such as "minimal", "maximal" or "product", we can obtain the transformation functions to recalibrate the combined p-values to satisfy the condition of p-values are uniform distributed under true null hypotheses. However, for other more complicated meta-analysis methods, the transformation function cannot be determined accurately leading to under- or over-estimation of significance, and ConReg-R can provide the polynomial function approximation for the unknown transformation.

Suppose for gene *i*, the p-values *p_ij _*(*j *= 1, 2, ..., *L*) follow the uniform distribution over (0, 1), then 1 - (1 - *p*_min_)*^L ^~ U*(0, 1) and , where *p*_min _= min(*p*_*i*1_, *p*_*i*2_, ..., *p*_*iL*_) and *p*_max _= max(*p*_*i*1_, *p*_*i*2_, ..., *p*_*iL*_). For the p-values from "product" method,  according to Fisher's method [19].

For each meta-analysis method, we simulated two data sets *Z*^0 ^= (*δ_ij _*)*, Z *= (*z_ij _*)(*i *= 1*, ..., n, j *= 1*, ..., r*) with *n*(*n *= 10000) genes and *r*(*r *= 10) repeats based on the formula as follows,

where *b_i _*(*b_i _*= 1, if *i *≤ *n*(1 - *π*_0_) and *b_i _*= 0 if *i > n*(1 - *π*_0_)) denotes the biological effect, both *δ_ij ~ _N*(0, 1) and *ε_ij _N*(0, 1) are the background noise. The individual p-values are computed from two-sample t-test and the combined p-values are calculated by *L*(*L *= 3) simulations.

To compare the results, we also included two other transformation methods, "square" and "square root". All methods are listed in Table 1.

**Table 1:** Combined *p*-values methods [18].

Method	Formula	Transformation
Min	*p*_min _= min(*p*_*i*1_, *p*_*i*2_, ..., *p*_*iL*_)	1-(1-*p*_*min*_)^*L*^
Max	*p*_man _= max(*p*_*i*1_, *p*_*i*2_, ..., *p*_*iL*_)	
Square		
Sqroot		
Prod		

**Figure 5:**
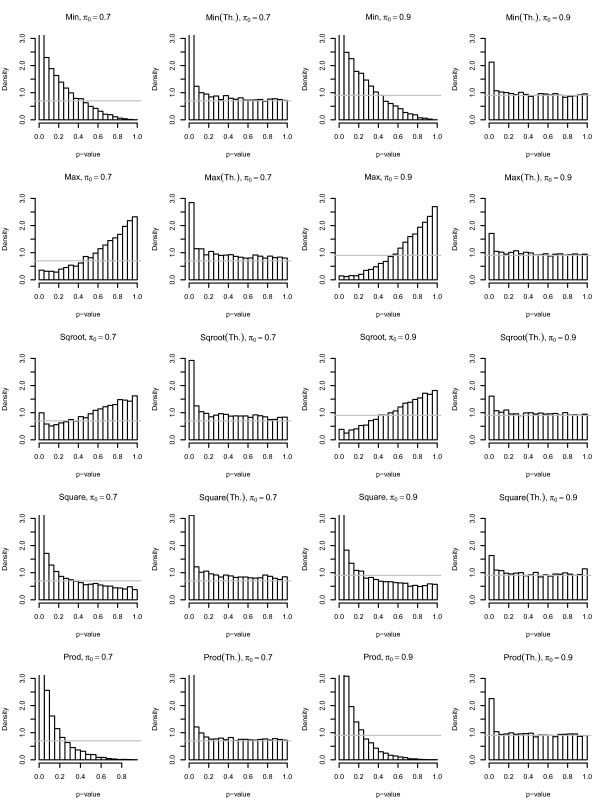
**The density histograms for "Min", "Max", "Sqroot", "Square" and "Prod" datasets at *π*_0 _= 0.7 and *π*_0 _= 0.9**. (Th.) indicates the density histograms for each method after theoretical transformation. The gray horizontal line indicates the *π*_0 _for each plot.

The two p-value histograms for each *π*_0 _= 0.7 and *π*_0 _= 0.9, and for each of five different methods are plotted in Figure 5. It can be seen from Figure 5 that the p-value histograms after theoretical transformation have constant frequency after 0.5 and the p-value density near 1 indicates the . However, the p-value histograms from "Min", "Square", "Prod" shifted towards 0 and the p-value histograms from "Max", "Sqroot" shifted towards 1.

**Figure 6:**
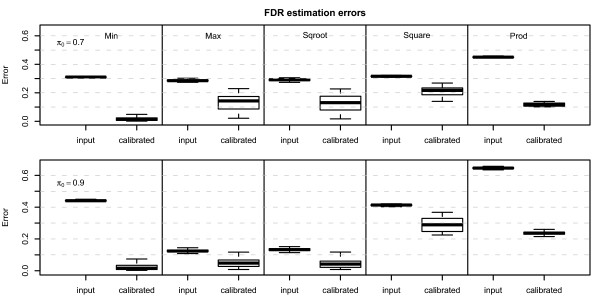
**Boxplots of FDR estimation errors for 100 simulations of "Min", "Max", "Sqroot", "Square" and "Prod" datasets at *π*_0 _= 0.7 and *π*_0 _= 0.9 before (input) and after applying ConReg-R (calibrated)**.

ConReg-R used the above combined p-values as input and the results are shown in Additional File 2, Figure S2 (*π*_0 _= 0.7) and Additional File 1, Figure S3 (*π*_0 _= 0.9). From Figure S2 and Figure S3, the regression curves are monotonic concave functions for "Min", "Square", "Prod" and monotonic convex functions for "Max", "Sqroot". The histograms after applying ConReg-R are also very similar to the theoretical transformed p-value histograms. The FDR estimation improved significantly after applying ConReg-R. It shows that the estimated FDR after applying ConReg-R is more likely to be the real FDR. The results of using our procedure for 100 repeated simulations are summarized in Figure 6. The FDR estimation errors after applying ConReg-R are significantly less than those obtained without applying ConReg-R.

### Yeast Environmental Response Data

Yeast environmental stress response data generated by [20,21] for nearly 6000 genes of yeast (*S. cerevisiae*) was aimed at understanding how yeast adopts or reacts to various stresses present in its environment. We selected 10 datasets: (1) Heat shock from 25°*C *to 37°*C *response; (2) Hydrogen peroxide treatment; (3) Menadione exposure; (4) DTT exposure response; (5) Diamide treatment response; (6) Hyper-osmotic shock response; (7) Nitrogen source depletion; (8) Diauxic shift study; and, (9-10) two nearly identical experiments on stationary phase. We used Limma (Linear Models for Microarray Data) [12] package in R to compute p-values for responsiveness of genes for each dataset.

The p-value distribution for each dataset is shown in Additional File 2, Figure S4. As can be seen in Figure S4, the majority of the p-value histograms do not have similar frequency after *p *= 0.5, and the density near *p *= 1 is less than *π*_0 _= 0.5. This implies that the p-values were under-estimated and the number of significantly responsive genes under these environmental stresses should be less than observed. We applied ConReg-R on the p-values of each dataset. Our result shows that the histograms of recalibrated p-values obtained by applying ConReg-R are better than without recalibration, and *π*_0 _estimations are all above 0.5 (Figure S4).

We use a true positive set of 270 genes from [22] to compute true FDR (*FDR^r^*). This is the intersection of core environmental stress response genes obtained by co-regulation study in [21] and the yeast orthologs of *S. pombe *stress response genes. These 270 genes have been used as the true positive sets in other studies [23,24]. The true FDR is calculated based on this 270 gene list and we calculated the improvement of FDR estimation (*FDR_im_*) for each dataset after applying ConReg-R. The *FDR_im _*is defined as followed:

where  (respectively, ) is the estimated FDR by recalibration (respectively, input) p-values for gene *i*(*i *= 1 ... *n*); and  is the true FDR for gene *i*.

**Figure 7:**
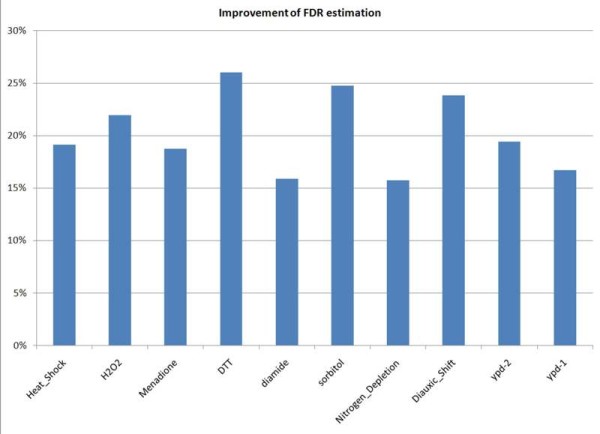
**Improvements in FDR estimation for yeast environmental response datasets**.

The improvements in FDR estimation for all 10 datasets are shown in Figure 7. After applying ConReg-R, FDR estimation improved by 15% to 25% which means that the FDR estimation will be closer to the real FDR.

**Figure 8:**
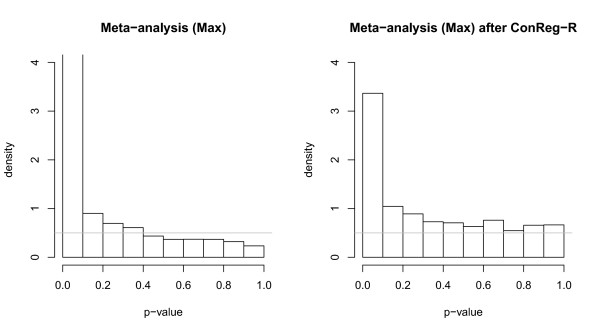
**The p-value density histograms for meta-analysis ("Max") before and after applying ConReg-R using yeast environmental response datasets**. The gray horizontal line indicates *π*_0 _= 0.5 for each plot.

We performed the meta-analysis for 10 datasets to detect the core environmental stress response genes using "maximal" method. The combined p-values are computed by the maximal p-values across 10 datasets, and then transferred to meta analysis p-values by transformation function in Table 1. The p-value density histograms for meta-analysis before and after applying ConReg-R are shown in Figure 8. The meta-analysis p-values show better distribution after first applying ConReg-R to each dataset and then perform the meta analysis. And FDR estimation improved by 38.5% after applying ConReg-R.

### Significance Analysis of Differential Expression from RNA-seq Data

The next-generation sequencing technologies have been used for gene expression measurement. In [25], the authors compared RNA-seq and Affymetrix microarray experiments and claimed that the sequencing data identified many more differentially expressed genes between human kidney and liver tissue samples than microarray data using the same FDR cutoff. In total, 11,493 significant genes were identified by RNA-seq (3380 more genes than Affymetrix), only 6534 (56.9%) genes were also identified by Affymetrix experiments. By checking the p-value histograms for RNA-seq dataset, we found that majority of p-values are very significant and its frequencies are very non-uniform for *p >*0.5. However, the p-value histogram for Affymetrix datasets is close to uniform for *p >*0.5 (Additional File 2, Figure S5).

**Figure 9:**
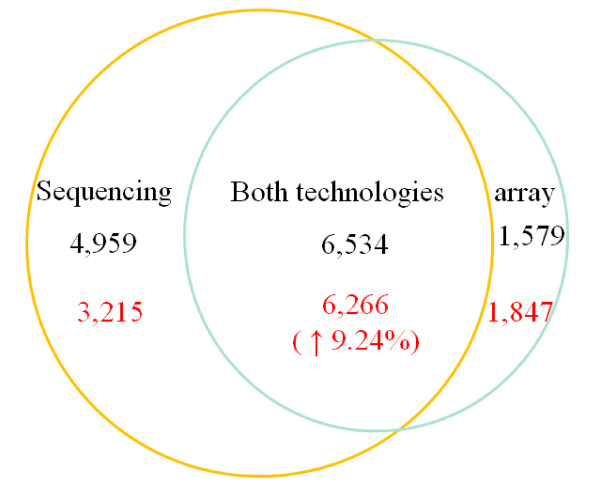
**A Venn diagram summarizing the overlap between significantly differentially expressed genes identified by sequencing (left circle) and microarray (right circle) technologies**. The numbers in black are the numbers reported in [25]. The numbers in red are the numbers after applying ConReg-R.

We applied ConReg-R to recalibrate the p-values obtained from RNA-seq datasets and re-estimated the FDR. We found 9481 significantly differentially expressed genes (only 1368 more genes than affymetrix) at *FDR ≤ *0.1%. Among them, 6266 genes (66.1%) were also identified by Affymetrix experiments. There is an increase of 9.2% overlap after application of ConReg-R (Figure 9). The FDR estimation is improved by 20% after applying ConReg-R if we used significant genes identified by affymetrix experiments as the true positive set.

## Discussion

ConReg-R focuses on the uniformity of p-values under null hypotheses and uses constrained polynomial regression to recalibrate the empirical p-value distribution to more well-defined p-value distribution. Therefore, the FDR estimation can be improved after the recalibration since the assumption of FDR estimation is that the input p-values should follow such an ideal empirical p-value distribution under null hypothesis. If the input p-values follow the properties of ideal empirical p-values distribution, the regression function tends to be diagonal line (i.e., *y *= *x*) and the p-values do not change considerably after recalibration.

Though our method is discussed in the context of global FDR control, it is equally applicable to the other FDR like controls such as local FDR. Our method does not provide any new FDR control, but inputs better calibrated p-values to the existing FDR estimators to improve their efficacy.

In ConReg-R, the cutoff of  and local minimization criteria can be changed for choosing the suitable *k *after checking the plots of  and . From the combined p-values simulation, the regression function may not fit the data well for the "Square" case. The fractional power, such as 1/2, can be added in the polynomial function to improve the fit.

The assumption that the regression function is convex or concave can be useful to deal with the imprecise p-values whose distribution is biased towards 1 or 0 respectively. These are the most common cases of the p-values being under-estimated or over-estimated. However, in some cases, the p-values can be under-estimated in one range of p-values and over-estimated in the remaining range of p-values. These p-value distributions may have peak or valley in the middle of the p-value range or even have multiple peaks. The regression function will then no longer be convex nor concave. Regression function to handle this situation is currently under study. Our ConReg-R can be generalized to an iterative weighted least squares method (e.g. decreasing weights from 1 to n). The weight program in the current version of the ConReg-R is assigning a weight of 1 for all p-values from 1 to k and a weight of 0 for the rest. Furthermore, different optimization schemes also need to be experimented. These will be explored in our future work. The distribution of p-values from multiple testing can be modeled by the mixture of uniform distribution and some other well-defined distribution such as Beta distribution [5]. The parametric recalibration method is under development. The discrete p-values from some nonparametric tests cannot be modeled by mixture model and new procedure should be explored to resolve this kind of problem.

## Competing interests

The authors declare that they have no competing interests.

## Authors' contributions

RKMK and KPC proposed the project. JL and RKMK developed the model, implemented the algorithm and wrote the paper. All the authors evaluated the results and approved the manuscript.

## Reviewers' comments

### Reviewer's report 1

Prof. Vladimir Kuznetsov, Bioinformatics Institute, A*STAR, Singapore

#### Review

Major: Summary. Mathematical part of the manuscript is major, but it is not convicted. Choosing the model (2) is not justified. The used model assumes a uniform distribution of the tail of FDR distribution is not correct due to experimental data; the most empirical FDR distribution exhibits the non-homogeneous fat tail as it was showed in Suppl. 1.

***Authors' response***: *Thanks for your comment. The p-value distribution under true null hypotheses follows the uniform distribution if the data satisfy all the assumptions of the hypothesis testing method *[4]*. However, the FDR distribution may not have similar property. The model (2) is based on the properties of the p-value distribution, but not on the FDR distribution. For experimental data, many factors influence those assumptions and the p-value distribution may not have uniformly distributed tail which is central to the paper. We clarified this in paragraphs 2, 3 & 4 in page 2*.

The final FDR distribution of combined p-value function is not be uniform "even under true null hypotheses", because it is a mixture distribution of the samples from different populations. If the samples are taken from significantly different distributions with different sample size and possible batch effect (not removed), does not allowed you to combine the datasets. If the samples are taken from the same distribution, why should not the p-value distributions be uniform (assuming that they were uniform in the individual datasets before merging)?

***Authors' response****: In combined p-values simulation, we assume p-values follow the uniform distribution for each dataset under true null hypotheses. For each gene, the overall p-value was obtained by combining its p-values across experiments using different combination methods which are typical non-parametric meta-analysis methods. The combined p-value distributions are not uniform which explains why another distribution is used to derive the overall p-value *[18]*. We clarified this in paragraphs 1 and 2 in page 9*.

Questionable statements

p.3

"Dependence among the test statistics is one of the major factors". It would be nice if you give examples of the test statistics you are mentioning.

***Authors' response****: We added the "t-statistic" as an example in text (paragraph 1 in page 3)*. "large p, small n problem". Could you describe the meaning this problem?

***Authors' response****: "large p, small n" problem is the problem that the number of variables (p) is much bigger than sample size (n) which always occurs in gene expression studies. We added one reference *[11]* for this problem (paragraph 2 in page 3). The n and p used in this context are different from that used in the discussion throughout the paper*.

"ConReg-R first maps the observed p-values to uniformly distributed p-values". From the context of this paragraph it is unclear where the set of 'uniformly distributed p-values' is obtained from.

***Authors' response****: We changed to "predefined uniformly distributed p-values" (paragraph 3 in page 3)*.

p.4

"Let pi' denote the ideal p-values". How did you decide that the ideal distribution of the p-values is a linear function, as defined in eq2 and is data- and test-independent?

***Authors' response***: *is the "ideal" p-values. By our definition in equation 2, their distribution is exactly uniform distribution. The "ideal" p-values are predefined by equation 5 and not related to any data or test. We clarified this in paragraph 3 in page 4*.

"the recalibration functions f() between p':(1..k) and p:(1..k) and apply it to all input p-values (i.e p:(1..n))". However, you mentioned before (p.3) that the distribution of p:(1..n) is a mixture of functions. Hence, how technically valid is mapping the whole set p:(1..n) with a function empirically estimated on a limited subset p:(1..k) ? How do you ensure that the parameters of mapping function f() estimated on the subset (1..k) are also valid in the subset (k+1..n) ?

***Authors' response****: This is the reason that we emphasized throughout the manuscript that our procedure is extrapolative in nature. The constraints and the choice of k in our procedure help the extrapolation reasonable as shown in our simulations and real data. This is discussed in the methods section. However, we cannot guarantee that the mapping function is 100% valid for all the data*.

"By Stone-Weirstrass theorem, polynomial functions can well approximate (a small typo here) any continuous functions". Before you defined p:(1..n) as a discrete set. How do you ensure that the function defined on this set continuous? Why quadratic programming is chosen for polynomial approximation, but not linear? Why not other function, e.g. cubic splines, sum of exponents with least squares fitting?

***Authors' response****: Since p-values are continuous, the mapping function from p-values to p-values should be continuous function. The discrete set p(1..n) is only a sample set from p-values. We used quadratic programming because the constraints are needed in approximation. We clarified it on last three paragraphs in page 4. We agree that different optimization procedures also can be chosen. But they are subject of our future study*.

p.5

"where ai,(i = 1,...,l) are l randomly generated numbers following U(0,1)". Why do they need to be generated randomly from U(0,1)? Since eq9 and eq10 are solved for beta under A * beta >= *b*, the solution beta depends on the random constrains A. "we add a sufficiently small positive value (delta(Q) = 10-10) to the diagonal of Q". Since Q = XXT, this is equivalent to delta(X) = sqrt(delta(Q)) = 10^-5^.

***Authors' response****: Since the range of × is *(x_k_, 1), we generated a_i _from U(0,1) to guarantee the constraint A * beta >= *b is valid in (0,1). The points of constraints can also be chosen uniformly throughout the range [0,1]. As we chose 10000 points for constraints, the uniform selection or random selection may not make significant difference. We clarified it on paragraph 3 in page 5. The small positive value δ = 10^-10 ^is not the determinant value of Q. we changed the notation to λ (paragraph 2 in page 6)*.

p.6

"the polynomial function may not accurately fit the data due to the limitation of the polynomial maximal power (usually set the maximal power t = 10)". How do you ensure that your approximating polynomial is not over-parameterized?

***Authors' response****: Since we approximate the polynomial function with monotonic convex/concave constraints, the approximation is not over-parameterized. In *Additional File 2, *figures S2 and S3, the quadratic and cubic polynomial functions were well fitted by a polynomial of t = 10*.

"estimated pi0(k) may oscillate near the real pi0". What is the source of the oscillations? Are they periodic?

***Authors' response****: The oscillation is due to the extrapolation may be unreliable if less number of p-value are used for regression. It may not be periodic. To better reflect it, we changed the word to "fluctuate". We clarified it on paragraph 4 in page 6*.

"the k closest to 1 that gives stable estimate". What is the definition of stability in this context? What numerical criterion do you use to detect the stability?

***Authors' response****: We used error **to define the stability of the estimation, smaller error **implies more stable estimate. In results, we used error **< 0.05 to detect the stability. We clarified it on paragraph 1 in page 7 and paragraph 2 in page 8*.

p.7

"As can be seen in the panels B and D in Figure 2". On Figure 2 the panels are not named. On Figure 3 all the axes need to be labeled and legends for the blue and the red lines should be added. Overall, the description of the results presented on Figure 3 is not comprehensible.

***Authors' response****: We changed "panels B and D" to "plots B and D" (paragraph 3 in page 7) and updated in *Figure 2*. We added the labels and legends for *Figure 3.

p.8

"Combined p-values simulation". The whole subsection needs to be described in the "Methods" section, since it introduces novel numerical techniques. In this subsection when you state "FDR improved by XX%", it could be good to show the particular FDR values before the improvement and after the improvement as well. The p-value distributions for the sets of experimental data would better be shown in the paper, rather than in supplementary materials.

***Authors' response****: The methods section described the ConReg-R method and the combined p-value simulation subsection shows the simulation result by applying ConReg-R. So we think it is suitable in the result section. The particular FDR values before and after applying ConReg-R have large numbers and it is not possible to summarize by one table. So we used improvement percentage to show the efficacy of ConReg-R. The Figure S1 now becomes *Figure 5*in the paper*.

"In many analyses ...the product of p-values will be appropriate". Where do these examples come from? Especially, since *p*1 * *p*2 <*min*(*p*1*, p*2), it's not logical that the p-value of significance across, at least, half of the experiments is lower than the p-value in all the experiments.

***Authors' response****: The product of p-values is based on Fisher's method for combining independent tests of significance (reference *[19]*). The combined p-values for product and minimal method are not comparable before transformation*.

"The distribution of combined p-values will not be uniform even under true null hypotheses". If the samples are taken from significantly different distributions (i.e the batch effect was not removed), what allowed you to combine the datasets? If the samples are taken from the same distribution, why should not the p-value distributions be uniform (assuming that they were uniform in the individual datasets before merging)?

***Authors' response****: Please see our response to the 2nd comment*.

### Reviewer's report 2

Prof. Philippe Broet, JE2492, University Paris-Sud, France

This reviewer provided no comments for publication.

### Reviewer's report 3

Prof. Hongfang Liu, Department of Biostatistics, Bioinformatics, and Biomathematics, Georgetown University Medical Center, NW, USA

This reviewer provided no comments for publication.

## Supplementary Material

Additional file 1 **The R functions for ConReg-R**. This file contains the R functions for ConReg-R. Click here for file

Additional file 2 **Supplementary figures**. This file contains supplementary figures: Figure S1 to S5. Click here for file
