# Supplementary material for: ConReg-R: Extrapolative recalibration of the empirical distribution of p-values to improve false discovery rate estimates
Source: Biol Direct. 2011 May 20;6:27. doi: 10.1186/1745-6150-6-27 (PMC3130718; doi:10.1186/1745-6150-6-27)
Supplement: Additional file 1 — The R functions for ConReg-R. This file contains the R functions for ConReg-R. [file 1745-6150-6-27-S1.PDF]

```
##############################################################################################

# ConRegR: Constraint Regression Recalibration for p values

# The main function is conregr(p) that takes any p-vlaues as input and output the recalibration p-values.

# Copyright (C) 2010

# Authors:        Juntao Li, Puteri Paramita

# Maintainer:     Juntao Li <lij9@gis.a-star.edu.sg>

# Usage Restrictions: This function is only used for academic research.

#

##############################################################################################


library(class)

library(e1071)

library(quadprog)


##############################################################################################

conregr = function(p,power=10,de=1,kmin=NULL,k = seq(0.01,1,0.01), ymad_pi0 = NULL,

                 ymedian_pi0 = NULL,mad_cutoff=0.05,flag=NULL,c=0.0001)

##############################################################################################

# inputs #

# p: the input p-values

# power: the max power used for polynomial regression, default is 10.

# de: the denominator  of the fractional power, default is 1.

# Kmin: the best k used for regression, default is NULL.

# k: the search set of k, default is from 0.01 to 1 and interval is 0.01.

# ymad_pi0: the mad of pi0 estimation, default is NULL.

# ymedian_pi0: the median of pi0 estimation, default is NULL.

# mad_cutoff: the cutoff of mad of pi0 estimation, default is 0.05.
```

```
# flag: the convex flag, default is NULL.
# c:  the constant adding in D's diagonal, default is 0.0001.
#
# outputs #
# kmin: the best k used for regression.
# p_input: the input p values.
# p_adj: the output  recalibration p-values.
# pi0: the pi0 estimation.
##################################################################################
{
  p.sorted <- sort(p,decreasing=TRUE,index.return=TRUE)
  p = p.sorted$x
  if(is.null(kmin))
  {
    for (i in 1:length(k))
    {
      fit = quadprog_reg(p,k[i],power=power,m=de)
      ymad_pi0[i] = fit$mad_pi0
      ymedian_pi0[i]= fit$median_pi0
    }
    kmin = Find_Mink(k,ymedian_pi0,ymad_pi0,cutoff=0.01,mad_cutoff)
  }
  miny = quadprog_reg(p,kmin,power=power,m=de)
  return(list(kmin=kmin, p_input = p, p_adj = miny$p_adj,pi0=ymedian_pi0))
}

##################################################################################
#Following is the function to find peaks and transitions.
```

```
#I/P is a seies of numbers

#O/P is a series of 0,1,and2. 0 means no peak/transition. 1 for a transition from low to high value or high to low value. 2 for a real peak i.e. two immediate transitions.

#--------------------------------------------------------------------------------

# funkce pro automaticke oznaceni piku ve spektru (peaks)

# autor Brian Ripley

# span has to be odd number


peaks<-function(series,span=3)

{

  z <- embed(series, span)

  s <- span%/%2

  #v<- max.col(z) == 1 + s #From Ripley


  #v=1 for simple transition and v=2 for real peaks i.e. transitions on both sides: This is by RK Murthy

  vF<- max.col(z,ties.method="first") == 1 + s ; vL<- max.col(z,ties.method="last") == 1 + s ;v = vL+vF


  result <- c(rep(0,s),v) #FALSE was replaced by 0 by RK Murthy

  result <- result[1:(length(result)-s)]

  result

}


################################################################################

Find_Mink<-function(k,median_pi,mad_pi,cutoff=0.01,mad_cutoff=0.05)

{

  yyii=NULL

  for(i in 1:length(k))
```

```
 {
   #the value below (mad)subject to change
   if(mad_pi[i]<mad_cutoff& median_pi[i]<=1 ) #if(mad_pi[i]<0.05& median_pi[i]<=1 )
   {
       yyii=rbind(yyii,cbind(k[i],median_pi[i]))
   }
 } # (k) value to be used
yy=yyii[,2]
yyy=-1*yy
if(length (yy) == 0){kmin = 1}
if(length (yy) <2){kmin = yyii[1,1]}
if(length (yy)==2){kmin=yyii[which(yy==min(yy,na.rm=T)),1]}
if (length (yy)>2){
           valley=peaks(yyy,3)
           peak=peaks(yy,3)
    for(i in 1:length(peak)){if(peak[i]==2){peak[i]=3}}
           v=peak+valley
           ee=(which(v!=0))
           if(any(valley==2)&(length(ee)>1)){
                       delta = matrix(1,nrow = (length(ee)-1), ncol = 1)
                       for (i in 2:length(ee)){delta[i-1]=(yyii[ee[i-1],2]-yyii[ee[i],2])}
                       d=which(abs(delta)>cutoff)
                       if(length(d)== 0){ d=which(abs(delta)>0)}
                       #d=which(abs(delta)==max(abs(delta)))
                       kk=matrix(0,nrow = (length(d)), ncol = 1)
         for(i in d[1]:d[length(d)]){
                       if(delta[i,1]>0){ kk[i]=yyii[ee[i+1],1]}
                       if(delta[i,1]<0){ kk[i]=yyii[ee[i],1]}
```

```
                    }
                    kmin=kk
             }
             else{kmin=yyii[(which(yy==min(yy,na.rm=T))) ,1]}

 }
 if(length(kmin)>1){kmin=max(kmin,na.rm=T)}
 if(length(kmin)==0){kmin=1}
 return(kmin)
}
################################################################################
quadprog_reg = function(p1,k,power,m=1,flag=NULL,c=0.0001)
# p1 is the input p-value and k is the percentage for regression
# power is the regression maximum power, and m is the denominator of power
# flag is the convex flag and c is the constant adding in D's diagonal
{
 n = length(p1)
 p_1 = p1[1:floor(n*k)]
 p = NULL
 for(i in 1:floor(n*k))
 {
  p[i] = 1-((i)/n)
 }
 if(is.null(flag))
 {
  convex = 0
  for(i in 1:floor(n*k))
  {
```

```
    if(p[i]>p_1[i])
    {
      convex = convex +1
    }
  }
  flag = -1
  if (convex < floor(n*k)/2)
  {
    flag = 1
  }
}
pa = runif(n)
cX = c(p_1^0)
cA = c(rep(0,2*n))
cpwr = NULL
for(i in 1:(m*power))
{
  cX = c(cX,p_1^(i/m))
  cpwr = c(cpwr,p1^(i/m))
  cA = c(cA,(i/m)*pa^(i/m-1))
  cA=c(cA,flag*(i/m)*(i/m-1)*pa^(i/m-2))
}
X = matrix(cX,nrow = floor(n*k), ncol = m*power+1, byrow = F)
A = matrix(cA,nrow = 2*n, ncol = m*power+1, byrow = F)
pwr = matrix(cpwr,nrow = n, ncol = m*power, byrow = F)
Y = matrix(c(p),nrow = floor(n*k), ncol = 1, byrow = F)
D <- crossprod(X)
for(i in 1:(m*power+1)){ D[i,i] =D[i,i]+c}
```

```
  d <- crossprod(X, Y)
  b <- rep(0,2*n)
  de = eigen(D,only.values=F)$values
  if(length(de[de<(2e-17)])>0) return(list(optk = 2, optpi = 2,pi0_new=2))
  obj = solve.QP(D, d, t(A), bvec=b)
  a0 = obj$solution[1]
  a = NULL
  for(i in 1:(m*power))
  {
    a = c(a,obj$solution[i+1])
  }
  reg_pp=pwr%*%a + a0
  a = a/sum(a)
  reg_p = pwr%*%a

  pi0 = NULL
  for(i in 1:n) pi0[i] = i / (n*(1-reg_p[i]))

  return(list(p_k = p_1, p_unif = p, p_adj = reg_p,p_reg=reg_pp,
            median_pi0 = median(pi0), mad_pi0=mad(pi0)))
}
```
