# Supplementary material for: ConReg-R: Extrapolative recalibration of the empirical distribution of p-values to improve false discovery rate estimates
Source: Biol Direct. 2011 May 20;6:27. doi: 10.1186/1745-6150-6-27 (PMC3130718; doi:10.1186/1745-6150-6-27)
Supplement: Additional file 2 — Supplementary figures. This file contains supplementary figures: Figure S1 to S5. [file 1745-6150-6-27-S2.PDF]

Supplementary figures

Figure S1

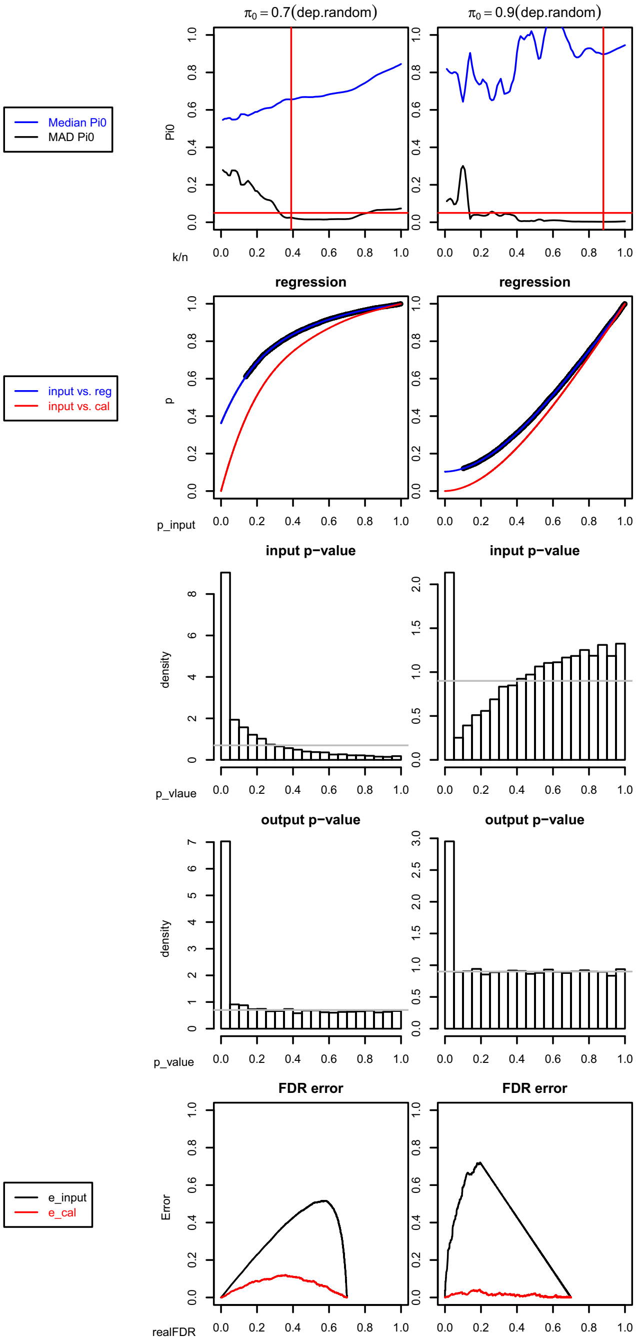

The procedural steps for the independent and dependent datasets with random dependent effect at $\pi_0 = 0.7$ and $\pi_0 = 0.9$. The plots in first row show the $\hat{\pi_0}$ and $e_{\hat{\pi_0}}$ at different k/n. The blue curve indicates $\hat{\pi_0}$ and the black curve indicates $e_{\hat{\pi_0}}$, the red horizontal line indicates the cutoff of $e_{\hat{\pi_0}}$ (here we used 0.05), the red vertical line indicates the choice of k/n at which locally minimized $\hat{\pi_0}$ under $e_{\hat{\pi_0}} < 0.05$ is obtained. The plots in second row show the regression

procedure. The black thick curve indicates the $(p_i, p_i'), i = 1, \ldots, k$ and the blue curve is the regression line $h_k(.)$, and the red curve is the regression line $f(.)$ after transformation. The plots in third and fourth row show the p-value histograms before and after applying ConReg-R and the gray horizontal line indicates the π_0. The plots in last row show the FDR estimation errors between real FDR and the FDR estimated by p-values before (black) and after applying ConReg-R (red).

Figure S2

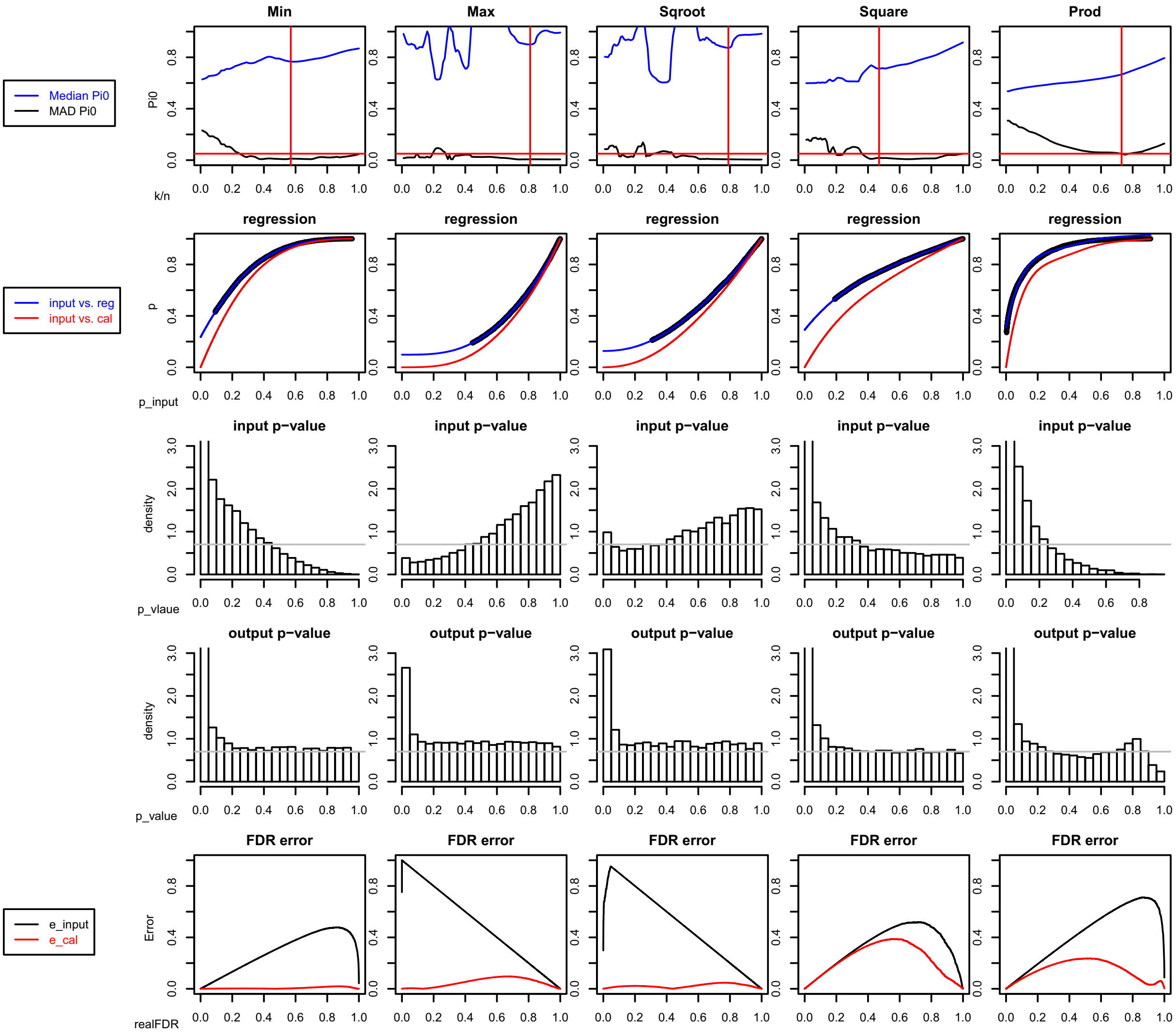

The procedure details for “Min”, “Max”, “Sqroot”, “Square” and “Prod” datasets at $\pi_0 = 0.7$.The detail description for plots in each raw is same as Figure S1.

Figure S3

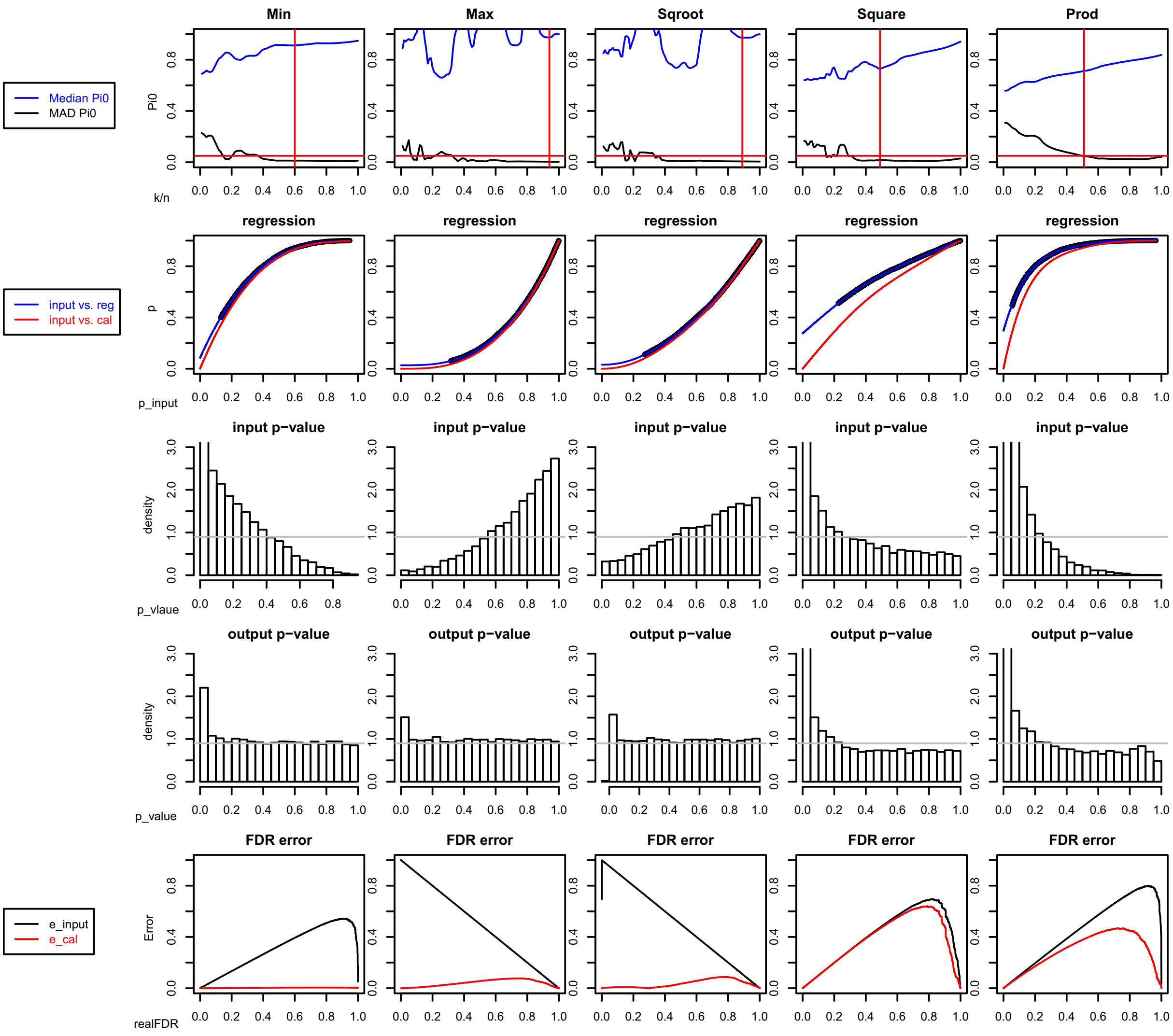

The procedure details for "Min", "Max", "Sqroot", "Square" and "Prod" datasets at $\pi_0 = 0.9$. The detail description for plots in each raw is same as Figure S1.

Figure S4

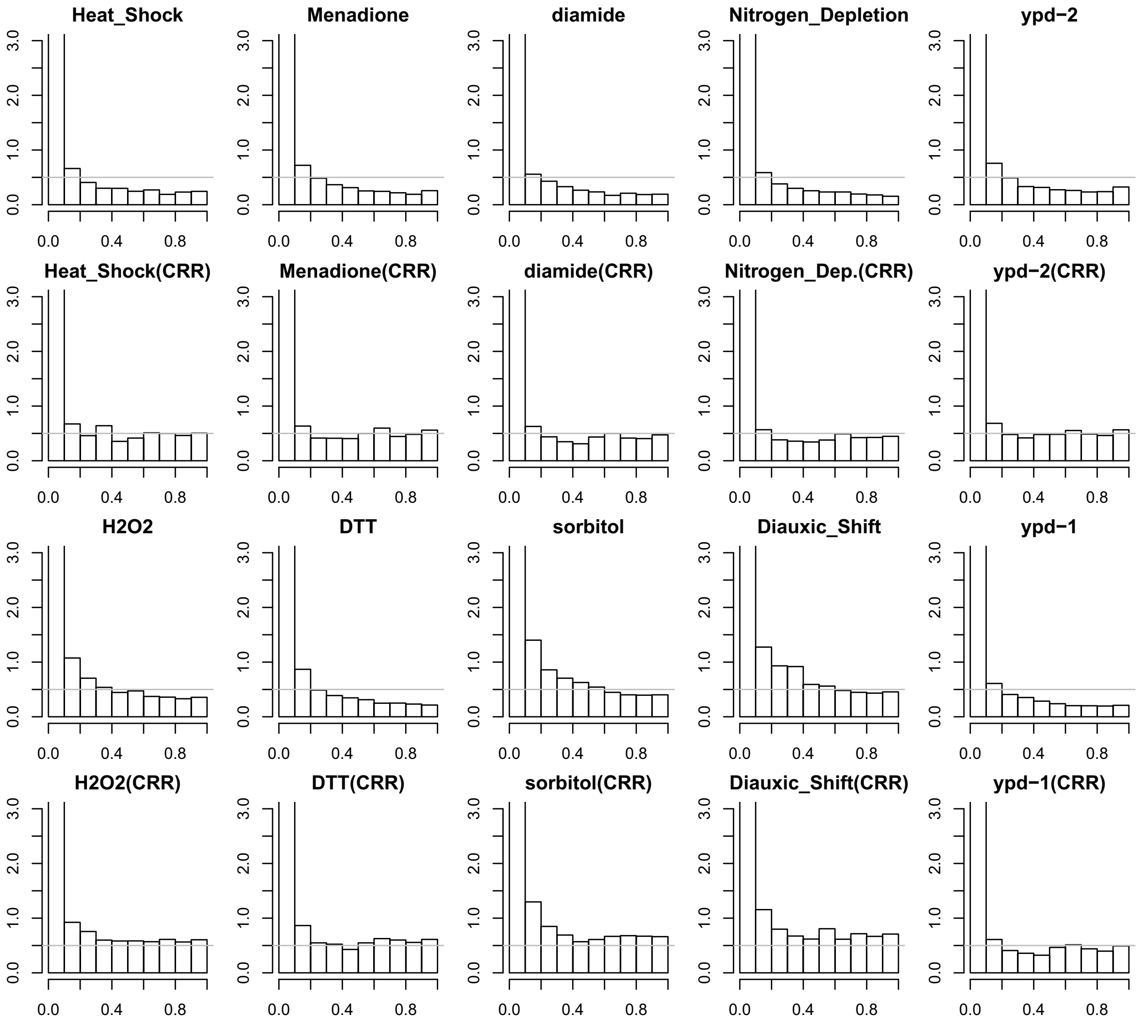

The p-value density histograms for 10 stress response data sets. (CRR) indicates the re-estimated p-values after ConReg-R. The gray horizontal line indicates $\pi_0 = 0.5$ for each plot.

Figure S5

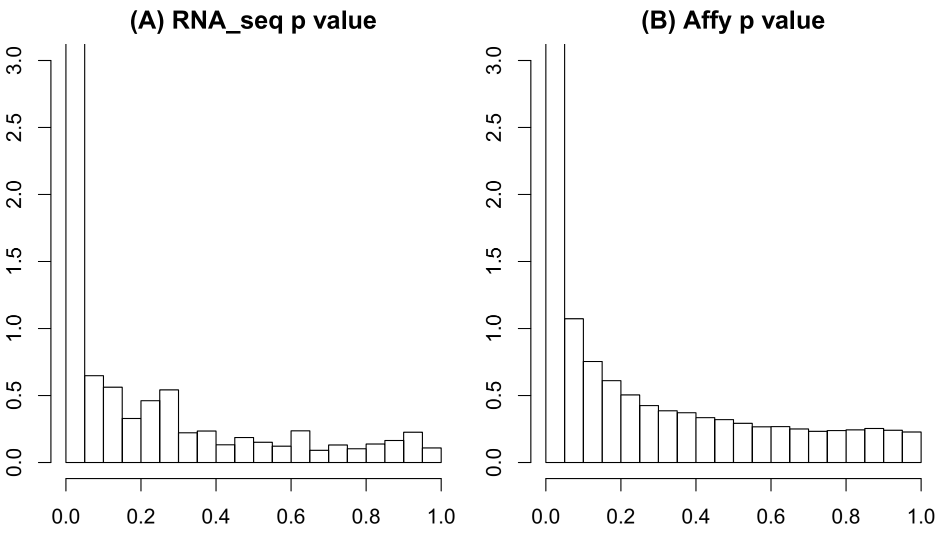

p-value density histograms for RNA-seq and Affymetrix datasets.
